# Decrease in choroidal blood flow after half and one-third dose verteporfin photodynamic therapy for chronic central serous chorioretinopathy

**DOI:** 10.1186/s12886-021-01980-w

**Published:** 2021-05-31

**Authors:** Shun Kumashiro, Seiji Takagi, Takashi Itokawa, Akiko Tajima, Tatsuhiko Kobayashi, Yuichi Hori

**Affiliations:** Department of Ophthalmology, School of Medicine, Toho University, 6-11-1 Omori-nishi, Ota-ku, Tokyo, 143-8541 Japan

**Keywords:** Central serous chorioretinopathy, Laser speckle flowgraphy, Choroidal blood flow, Central choroidal thickness

## Abstract

**Background:**

The effect of various reduced doses of verteporfin photodynamic therapy (PDT) on choroidal blood flow in chronic central serous chorioretinopathy (CSC) remains unclear. Therefore, this study aimed to evaluate choroidal blood flow after half-dose PDT (1/2PDT) and one-third dose PDT (1/3PDT) with verteporfin for chronic CSC using laser speckle flowgraphy and spectral-domain optical coherence tomography.

**Methods:**

Twenty-seven eyes of 27 patients with serous retinal detachment (SRD) caused by chronic CSC for more than 6 months were included in this study. Patients were divided into the 1/2PDT (*n* = 12; January 2018 to July 2019) and 1/3PDT (*n* = 15; August 2016 to December 2017) groups based on the treatment period. The best-corrected visual acuity (BCVA), central retinal thickness (CRT), central choroidal thickness (CCT), and mean blur rate in the macular area (m-MBR) and optic nerve head (ONH-MBR) were obtained using laser speckle flowgraphy and evaluated at baseline (pre-treatment), and 2 weeks, 1 month, 3 months, and 6 months after treatment.

**Results:**

We found that SRD disappeared after 1 month in 92 and 93% of patients’ eyes in the 1/2PDT and 1/3PDT groups, respectively. Recurrence of SRD was observed in one eye at the 6-month follow-up after 1/2PDT and two eyes at the 3-month follow-up after 1/3PDT. No significant improvement was observed in baseline BCVA in the 1/3PDT and 1/2PDT groups. The average m-MBR against baseline significantly decreased at 2 weeks and 1, 3, and 6 months in the 1/2PDT group. The average m-MBR against baseline decreased significantly only at the 2 weeks follow-up in the 1/3PDT group. The average rate of change in the CCT against baseline decreased significantly throughout for up to 6 months in the 1/2PDT group and for up to 3 months in the 1/3PDT group. No significant fluctuation was observed in the ONH-MBR.

Conclusions: Here, PDT significantly affected choroidal blood flow depending on the verteporfin dose in chronic CSC.

**Trial registration:**

This trial was retrospectively registered (UMIN000026850; Approval date 03/04/2017).

## Background

Recent advancements in retinal imaging technology have enabled novel methods of investigating choroidal pathology in retinal diseases. The choroid performs highly important physiological functions such as providing vascular supply, nutritional support, and oxygen to the outer retina [1].

Central serous chorioretinopathy (CSC) is one of the most common retinal diseases, which presents with central serous chorioretinopathy, serous retinal detachment (SRD) at the posterior pole, and causes blue vision and metamorphopsia, and (especially) micropsia [2].Although the clinical course of CSC is usually benign, 30 to 50% of patients experience repeated recurrence, and some of these patients develop chronic CSC, which results in poor visual acuity [3, 4].The pathophysiology of acute and chronic CSC is not fully understood, although several studies have revealed that retinal pigment epithelium (RPE) and choroidal dysfunction could play an important role [5, 6] Gass suggested that a focal increase in the permeability of the choriocapillaris layer was the primary cause of damage to the overlying RPE in patients with CSC [6]. Focal leakage on fluorescein angiography (FA) and dilated choroidal vessels and focal choroidal vascular hyperpermeability on indocyanine green angiography (ICGA) are suggestive of the increase in choroidal hydrostatic pressure and defects in tight junctions of the RPE cells in CSC [7].

Photodynamic therapy (PDT) with verteporfin, which was introduced as a promising therapeutic approach for CSC, is based on occlusion and remodelling of the choroidal capillaries and choroidal vascular, which decreases the hyperpermeability of the dilated choroidal vessels [8, 9]. Although these treatments yielded good results, complications such as RPE atrophy, RPE tear, secondary choroidal neovascularization (CNV), and choriocapillaris ischemia occurred after PDT [10–12].

To minimise these side effects and to achieve maximum efficacy, research has been conducted in recent years. Thereafter, studies have reported the efficacy of reduced doses of verteporfin PDT in minimising the angiogenetic complications responsible for severe visual dysfunction in CSC. Studies on half-dose PDT (1/2PDT) have shown favourable results with comparable effects and fewer side-effects than those with full-dose PDT [13, 14]. Moreover, 30%-PDT has been shown to reduce retinal thickness, with a good FA–based improvement rate for acute CSC [15].

Laser speckle flowgraphy (LSFG), a commercially available device, provides a non-invasive quantitative method of determining ocular blood flow, including choroidal blood flow [16].Saito et al. reported a significant decrease in blood flow against the baseline in acute CSC [17]. However, the effect of various reduced doses of verteporfin PDT on choroidal blood flow in chronic CSC remains unclear.

The purpose of this study was to investigate the changes in the choroidal blood flow and thickness after 1/2PDT and one-third dose PDT (1/3PDT) in patients with chronic CSC.

## Patients and methods

The institutional review board of Toho University Omori Medical Center approved the protocol of this retrospective study review (approval number: 27–277). All patients provided informed consent for participation after they received an explanation of the nature and possible consequences of the treatment in accordance with the tents of the Declaration of Helsinki. This study was registered with the UMIN clinical trial registry (UMIN ID: 000026850).

### Patients

Consecutive cases of patients with chronic CSC who were admitted to Toho University Medical Center Omori Hospital were recruited between August 2016 and February 2019. All participants were Asian. All patients underwent basic ophthalmic examinations, including best-corrected visual acuity (BCVA), intraocular pressure (IOP), fundus examination, spectral-domain optical coherence tomography (SD-OCT), and LSFG. FA and ICGA were performed at the initial visit. BCVA was obtained using Landolt C charts. These values were subsequently converted to the logarithm of the minimum angle of resolution (logMAR) equivalent for statistical comparisons. The exclusion criteria included patients with a history of retinal photocoagulation, presence of CNV or other ischaemic retinopathies, a history of intravitreal injections of anti-vascular endothelial growth factors, and those taking medications such as corticosteroids, adrenergic agonists, or adrenergic antagonists.

### Diagnosis of chronic CSC

**Fig. 1:**
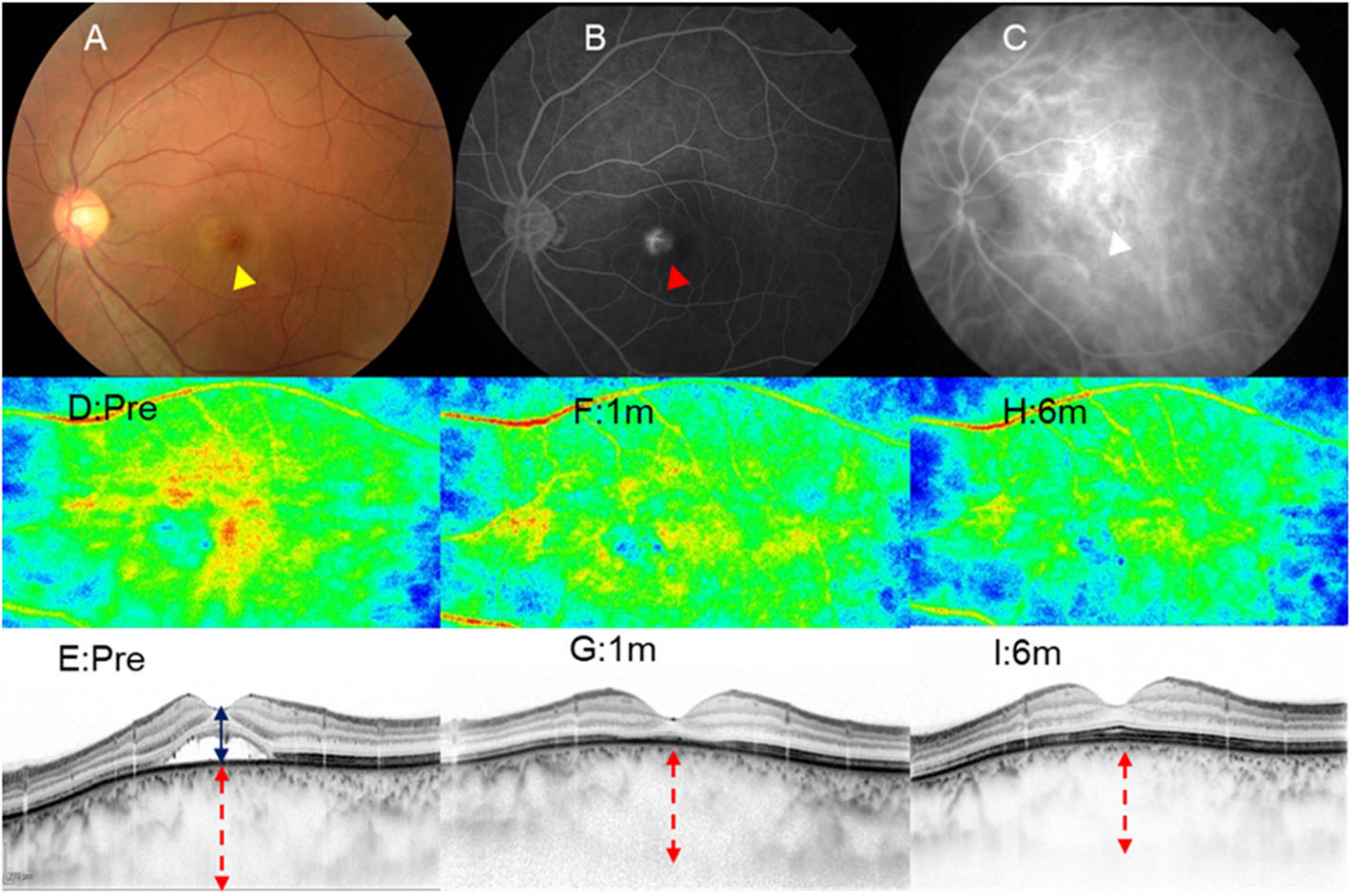
Representative case of a 42-year-old man with chronic central serous chorioretinopathy. Fundus images of the left eye obtained pre-treatment (a–c, d, e), 1 month (f, g), and 6 months after treatment (h, i), who were treated with half-dose verteporfin photodynamic therapy. **a** The fundus photograph shows serous retinal detachment with 1-disc-diameter size at the fovea (yellow arrow). **b** Early-phase fluorescein angiography (FA) shows leakage at the nasal side of the fovea (red arrow). **c** The middle-phase of indocyanine green angiography (ICGA) shows diffuse choroidal hyperpermeability at correspondence with leaking spots on FA (white arrow). **d-h** Laser speckle flowgraphy (LSFG) and horizontal sectional optical coherence tomography (OCT) images. **d**, **e** The red and yellow areas on LSFG represent a relatively high mean blur rate (MBR) at the macular area, which corresponds to the area of hyperpermeability on ICGA. OCT shows serous retinal detachment at the macula (blue double arrow) and a thickened choroid (red dash double arrow, 596 μm). **f**, **g** LSFG shows fewer red and yellow areas, which indicate a reduction in choroidal blood flow. Serous retinal detachment at the macula disappeared with choroidal thinning (558 μm) 1 month and 6 months (555 μm) after treatment

Patients were diagnosed with CSC based on clinical findings such as the presence of subretinal fluid (SRF) and macular choroidal vasodilatation on optical coherence tomography (OCT) imaging, fluorescein leakage below or near the central fovea of the macula on FA, and diffuse vascular permeability on ICGA. Figure 1 depicts a representative case of CSC.

Chronic CSC was defined as persistence of SRF for more than 6 months with no improvement after conservative treatment or expectation of spontaneous resolution.

Each case of CSC was divided into active and resolved eyes based on the presence of SRD in the macular area. The presence of any fluid was designated as active CSC.

### One-third and half-dose reduced photodynamic therapy

Patients were divided into the 1/3PDT group (verteporfin 2 mg/m^2^) and 1/2PDT group (verteporfin 3 mg/m^2^) according to the treatment period: those who underwent PDT between August 2016 and December 2017 received 1/3PDT, and those who underwent PDT between January 2018 and July 2019 received 1/2PDT.

The treatment parameters for PDT included infusion of verteporfin (2 mg/m^2^ or 3 mg/m^2^) verteporfin for 10 min, followed by activation with a diode laser of 689-nm wavelength (Visulas 690 S; Carl Zeiss Meditec, Dublin, California, USA) with a radiation dose of 50 J/cm^2^ for 83 s at the site of hyperpermeability depicted by ICGA. The PDT-irradiated area was determined using ICGA to identify the area of choroidal vasodilatation and congestion and area with extravasation in the macula.

### OCT measurements

SD-OCT (Spectralis; Heidelberg Engineering, Heidelberg, Germany) images were acquired before treatment (i.e. at baseline) and 2 weeks and 1, 3, and 6 months after treatment to analyse the retinal and choroidal thickness. For all patients, a 9 mm horizontal B-scan section through the central fossa was used for analysis. No tracking function was used, and the images were taken while checking the monitor for the thinnest part of the outer layer. CCT was obtained with enhanced-depth imaging-OCT. The requisite parameters on the vertical and horizontal scans were measured manually using a built-in calibre tool, and the values were averaged. CCT was defined as the distance between the line corresponding to Bruch’s membrane beneath the RPE and chorioscleral interface under the fovea.

### Laser speckle flowgraphy

The LSFG-NAVI™ (Softcare Co., Fukuoka, Japan) was used to acquire the LSFG images before treatment and 2 weeks and 1, 3, and 6 months after treatment. The determination of optic nerve head (ONH) circulation with LSFG has been described in detail previously [18].In LSFG, when the fundus is irradiated with an 830 nm long-wavelength laser, a random speckle pattern is formed by the interference of reflected and scattered light by blood cells. The rate of change is used to calculate the blood flow velocity. The faster the red blood cells move, the larger the blur rate in the speckle pattern becomes. The mean blur rate (MBR) is calculated from this blur change and is used as a quantitative measure of relative blood flow velocity.

Previous studies have reported that the MBR is an indicator of retinal blood flow. The original MBR values were continuously recorded at 118 frames within 4 s, followed by averaging the entire data set to synthesise a still image corresponding to the duration of one heartbeat. The average MBR was determined as the mean value of the synthesised MBR histogram during one heartbeat. An offline analysis software (LSFG Analyzer, version 3.0.47.0) combined all the images and transformed each pixel into a colour-coded map to which the calculated mean blur ratio was assigned, which is a quantitative indicator of the relative blood flow rate.

We calculated the MBR in the macular area (m-MBR) and ONH-MBR using the LSFG Analyzer software.

The ONH-MBR was measured to evaluate the retinal blood flow. The MBR in the macular area (m-MBR), which is the retinal avascular region, was used to evaluate the choroidal blood flow. The m-MBR was measured to evaluate the changes in the MBR: the relative MBR was calculated against the baseline and expressed as the rate of change in relative blood flow velocity [23]. LSFG was performed after the participants had rested for 10 min in a quiet room maintained at 24 °C. The MBR was evaluated by calculating the rate of change against the first baseline measurement.

### Measurement of other systemic and ocular parameters

Pulse rate, systolic blood pressure (SBP), and diastolic blood pressure (DBP) were measured before PDT. The mean arterial pressure (MAP) was calculated by the formula of MAP = DBP + 1/3 (SBP - DBP). Ocular perfusion pressure (OPP) was calculated as OPP = 2/3 MAP-IOP [23].

### Statistical analyses

All values were presented as the mean and standard deviation unless specified otherwise. The unpaired t-test and Chi-squared test were used to compare the characteristics of the two groups. The rate of change of m-MBR and ONH-MBR in each group was evaluated using the Steel test, and the Wilcoxon test was used to compare the rate of change of m-MBR and ONH-MBR in the 1/2PDT and 1/3PDT groups.

In addition, *p*-values < 0.05 were considered statistically significant. The JMP (version 11) statistical analysis software (SAS Institute, Inc., Cary, NC, USA) was used to analyse the data.

## Results

No systemic and ocular adverse events associated with verteporfin injection were observed throughout the study period. The baseline demographic and clinical data of the two treatment groups are summarised in Table 1. A total of 27 eyes from 27 patients with chronic CSC were considered, of which 15 eyes were treated with 1/3PDT and 12 eyes were treated with 1/2PDT. No significant differences were found in the sex (*p* = 0.78), baseline BCVA (1/2PDT, 0.13 ± 0.25; 1/3PDT, 0.21 ± 0.25; *p* = 0.35), duration of symptoms (1/3PDT, 18.4 ± 16.4; 1/2PDT, 18.8 ± 12.2; *p* = 0.59), CRT at baseline (1/2PDT, 334.2 ± 94.5; 1/3PDT, 275.1 ± 94.5; *p* = 0.06), CCT at baseline (1/2PDT, 451.2 ± 132.5; 1/3PDT, 452.2 ± 85.1; *p* = 0.88), and PDT spot size (1/2PDT, 2888.3 ± 550.5; 1/3PDT, 2558.9 ± 710.7; *p* = 0.22), except for age (1/2PDT, 51.5 ± 5.7; 1/3PDT, 46.3 ± 7.2; *p* = 0.04) between the two treatment groups. The preoperative values in the fellow eye were CRT (205.5 ± 26.1), CCT (360.3 ± 114.9), and m-MBR (8.48 ± 3.37) for 1/2 PDT and CRT (231.7.5 ± 73.5), CCT (408.1 ± 106.3), and m-MBR (8.71 ± 3.14) for 1/3 PDT. Preoperatively, CRT and CCT were significantly elevated in the affected eye (CRT: 1/2PDT [*p* = 0.0002] and 1/3PDT [*p* = 0.0013], CCT: 1/2PDT [*p* = 0.039] and 1/3PDT [*p* = 0.0007]).

**Table 1:** Baseline demographic and clinical data of the 1/2PDT and 1/3PDT group

	1/2group	1/3group	*P* value
Age	51.5 ± 5.7	46.3 ± 7.2	0.04
sex (male:female)	9:03	12:03	0.78
BCVA (logMAR BCVA)	0.13 ± 0.25	0.21 ± 0.25	0.35
duration of symptoms (months)	16.8 ± 12.2	18.5 ± 16.4	0.59
IOP (mmHg)	15.0 ± 1.8	13.5 ± 3.7	0.06
OPP (mmHg)	51.3 ± 7.5	52.3 ± 12.6	0.90
CCT (μm)	334.2 ± 94.5	275.1. ± 94.5	0.67
CRT (μm)	451.2 ± 132.5	452.2 ± 85.1	0.88
PDT spot size (μm)	2888.3 ± 550.5	2558.9 ± 710.7	0.22

*BCVA* best-corrected visual acuity, *IOP* intraocular pressure, *OPP* ocular perfusion pressure, *CRT* central retinal thickness, *CCT* central choroidal thickness, *1/2PDT* half-dose photodynamic therapy, *1/3PDT* one-third dose photodynamic therapy, *logMAR* logarithm of the minimum angle of resolution.

The other values were not significantly different compared with the affected eye.

Preoperative CRT was significantly elevated in the affected eye for both 1/2PDT (*p* = 0.0002) and 1/3PDT (*p* = 0.0013), although the others were not significantly different compared with the affected eye.

The disappearance rate of SRF 1 month after treatment was 92% (11/12 eyes) in the 1/2PDT and 93% (14/15 eyes) in the 1/3PDT group.

Recurrence was observed in one eye at the 6-month follow-up in the 1/2PDT group and two eyes at the 3-month follow-up in the 1/3PDT group over the 6-month follow-up period.

No significant improvement was observed in the BCVA compared with baseline (0.13 ± 0.25 and 0.21 ± 0.25, respectively) at 2 weeks (0.13 ± 0.21 and 0.19 ± 0.25, respectively), 1 month (0.13 ± 0.22 and 0.13 ± 0.23, respectively), 3 months (0.07 ± 0.19 and 0.12 ± 0.27, respectively) and 6 months (0.02 ± 0.19 and 0.13 ± 0.30, respectively) after PDT in the 1/2PDT and 1/3PDT groups.

**Fig. 2:**
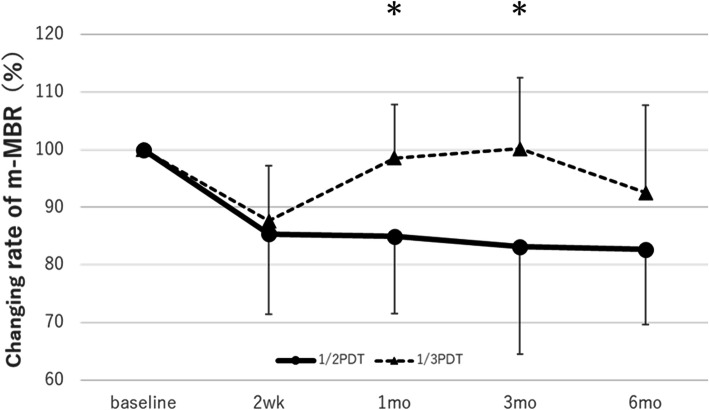
Change in the m-MBR before and after 1/2PDT and 1/3PDT in patients with chronic CSC. The change in the m-MBR was evaluated at baseline and 2 weeks, and 1, 3, and 6 months after 1/2PDT and 1/3PDT in patients with chronic CSC. The solid line represents the 1/2PDT group, and the dashed line represents the 1/3PDT group. The error bars represent the standard deviations of the participants. The asterisk represents significant differences between the 1/2PDT and 1/3PDT groups. * *p* < 0.05 (Steel test). 1/2PDT: half-dose verteporfin photodynamic therapy, 1/3PDT: one-third dose verteporfin photodynamic therapy, CSC: central serous chorioretinopathy, m-MBR: macular mean blur rate

Figure 2 depicts the time course of the rate of change in the m-MBR against baseline in the 1/2PDT and 1/3 PDT groups. In the 1/2 PDT group, the average m-MBR against baseline (100%) significantly decreased to 85.3% (*p* = 0.004), 85.0% (*p* = 0.037), 83.1% (*p* = 0.015), and − 82.7% (*p* = 0.015) 2 weeks and 1, 3, and 6 months after treatment, respectively. In the 1/3PDT group, the average m-MBR against baseline (100%) decreased to 87.2% (*p* < 0.001), 98.5% (*p* = 0.79), 100.2% (*p* = 0.99), and 92.5% (*p* = 0.14) 2 weeks and 1, 3, and 6 months after treatment, respectively. The rate of change in the m-MBR differed (statistically) significantly between the 1/2PDT and 1/3PDT groups at 2 weeks and 1 and 3 months after treatment.

### Foveal choroidal thickness

**Fig. 3:**
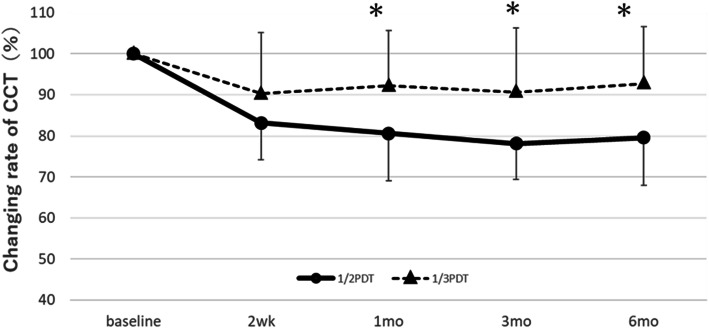
Rate of change in CCT before treatment after 1/2PDT and 1/3PDT in patients with CSC. The rate of change in CCT was evaluated before treatment and 2 weeks, and 1, 3, and 6 months after 1/2PDT and 1/3PDT in patients with CSC. The solid line indicates the 1/2PDT group, and the dashed line indicates the 1/3PDT group. The error bars represent the standard deviations of the participants. * *p* < 0.05 (Dunnett test). The asterisk represents significant differences between the 1/2PDT and 1/3PDT groups. * *p* < 0.05 (Steel test). 1/2PDT: half-dose verteporfin photodynamic therapy, 1/3PDT: one-third dose verteporfin photodynamic therapy, CSC: central serous chorioretinopathy, CCT: central choroid thickness

Figure 3 shows the time course of the rate of change in the CCT against baseline in the 1/2PDT and 1/3PDT groups. In the 1/2PDT group, the average rate of change of CCT against baseline (100%) significantly decreased to 83.1% (*p* < 0.001), 80.6% (*p* < 0.001), 78.1% (*p* < 0.001), and 79.6% (*p* < 0.001) 2 weeks and 1, 3, and 6 months after treatment, respectively. In the 1/3PDT group, the average of the rate of change of CCT against baseline (100%) decreased to 90.3% (*p* = 0.001), 92.2% (*p* = 0.001), 90.7% (*p* = 0.001), and 92.8% (*p* = 0.5) 2 weeks and 1, 3, and 6 months after treatment, respectively. The rate of change in CCT differed (statistically) significantly between the 1/2PDT and 1/3PDT groups 2 weeks and 1, 3, and 6 months after treatment.

The ONH-MBR showed no significant fluctuation at 106% after 2 weeks, 104% after 1 month, 101% after 3 months, and 102% after 6 months in the 1/2PDT group. The ONH-MBR showed no significant fluctuation at 99, 96, 96, and 99% after 2 weeks, 1 month, 3 months, and 6 months, respectively, in the 1/3PDT group.

Moreover, no significant change was observed at any time-point after treatment compared with baseline.

The OPP also showed no significant fluctuation at 98, 102, 103, and 110% for 2 weeks, 1 month, 3 months, and 6 months, respectively, in the 1/2PDT group and at 97,107, 102, and 104% for 2 weeks, 1 month, 3 months, and 6 months, respectively, in the 1/3PDT group.

We also compared the preoperative CRT, CCT, and m-MBR values between the affected and ipsilateral sides. The preoperative CRT and CCT values were significantly higher in the affected eye than in the ipsilateral eye. Other values were not significantly different.

We found a correlation between m-MBR and CCT (Spearman’s rank correlation test, *r* = 0.27, *p* = 0.0039).

## Discussion

Hyperpermeability of the choroidal blood vessels and the consequent increase in choroidal hydrostatic pressure comprise the current principal hypothesis to explain the pathophysiological mechanism of CSC [19]. ICGA depicts arterial filling delay and subsequent hyperpermeability in patients with CSC [20].We found that sub-foveal choroidal circulation decreased significantly after the administration of reduced doses of verteporfin PDT in patients with chronic CSC as depicted by the significant disappearance of SRF in the 1/2PDT and 1/3PDT groups on LSFG. There was no significant correlation between MBR and OPP, which suggests that systemic circulation did not affect the m-MBR.

To the best of our knowledge, this is the first report that investigated the alteration in choroidal blood flow using LSFG after PDT in chronic CSC using two clinically representative concentrations of verteporfin.

The salient feature of this study is that it evaluated the blood flow directly using LSFG. The majority of the MBR in LSFG is thought to be derived from choroidal blood flow. Moreover, the medium and large blood vessels of the choroid in the fovea are clearly visualised, which is the retinal avascular area. Therefore, the m-MBR is considered to reflect the pathological condition in CSC [21].Saito et al. reported that the mean rate of the change in m-MBR decreased significantly at 6 months with spontaneous remission of SRF and visual recovery in patients with acute CSC [17].

Damage to the vascular endothelium, hypoperfusion, and choroidal hyperpermeability due to the formation of free radicals associated with radiation is thought to be the therapeutic mechanism of action of PDT in CSC [9].Our finding, i.e. the notable decrease in m-MBR after PDT, supports this hypothesis. Interestingly, a significant decrease in the m-MBR in the 1/3PDT group was observed for only 1 month, in contrast to that in the 1/2PDT group, which was sustained for up to 6 months. Moreover, we found that the average time for the recurrence of SRD was shorter in the 1/3PDT group than in the 1/2PDT group. These results could indicate that the choroidal blood flow would be affected in a verteporfin dose-dependent manner. PDT with verteporfin is a promising therapeutic modality for CSC. However, it is accompanied by the risk of serious complications such as angiographic closure following treatment [11].Therefore, various regimens, including reduced dose, reduced power [22].reduced time [23].and reduced verteporfin [4, 24, 25], have been devised. Verteporfin concentrations affect normal choroidal vessels in a dose-dependent manner. Zhao et al. studied the treatment of acute CSC with PDT at verteporfin concentrations of 70, 60, 50, 40, 30, 20, and 10%, and found that the 30% dose of verteporfin (1.8 mg/m^2^) is the minimum required therapeutic dose [26].Moreover, they also reported that the 50% dose of verteporfin might be more effective in resolving SRF and fluorescein leakage with better visual outcomes in acute CSC than the 30% dose [27, 15].Our findings also support the notion that 50% of verteporfin is more beneficial with respect to the efficacy and safety of the modified PDT protocol.

Choroidal thickening is considered to be one of the most characteristic clinical signs of CSC and is associated with choroidal vascular hyperpermeability [28, 29]. The subfoveal choroid layer was thicker in the affected and fellow eyes in patients with CSC than in the normal control eyes [30]. Numerous OCT studies have reported structural changes after PDT. Maruko et al. reported that the subfoveal choroid thickness decreased significantly after a transient increase following 1/2PDT (verteporfin) [31]. Moreover, Kinoshita et al. reported that the decreased CCT might be attributed to the reduction in the dilation of the outer choroidal vessels in 1/2PDT (verteporfin) [32].Izumi et al. reported that the subfoveal intrachoroidal structure showed greater alteration than the choriocapillaris and medium choroidal vessels after 1/2PDT for CSC [33].

We found that the CCT decreased after 1/2 and 1/3PDT and was accompanied by a decrease in the m-MBR. We also found a positive correlation between CCT and m-MBR. This is consistent with reports of a significant correlation between CCT and m-MBR reduction after intravitreal bevacizumab injection in eyes with CSC [19].This may indicate that the decreased blood flow in the choroidal vessels resulted in choroidal thinning.

Furthermore, Iovino et al. observed a sustained decrease in central macular thickness and subfoveal choroidal thickness after PDT of chronic CSC, as well as a decrease in total choroidal area and luminal choroidal area at a 1-month follow-up interval [34].We reported that these results support the hypothesis that PDT targets large choroidal vessels and causes contraction and total choroidal volume reduction. Simultaneous evaluation of blood flow measurements using LSFG and choroidal structural changes using OCT and ICGA could explain and depict the pathophysiology of CSC. However, the relationship between m-MBR and CCT in retinal-choroidal disease is not uniform in other diseases. Although the choroid thickness increases in inflammatory diseases such as Harada disease [21]and acute zonal occult outer retinopathy [35], the m-MBR reportedly decreases owing to the status of circulation in the acute phase of inflammation.

We found no significant change in visual acuity after the resorption of the SRF, which could be attributed to the relatively longer disease duration (18.4 ± 16.4 months) in our chronic CSC population. The persistence of SRF in chronic CSC is thought to be associated with irreversible, progressive photoreceptor damage, leading to loss of visual function [36, 37].Clinical features such as later age of onset, cystoid macular degeneration, CNV, and disruption of the ellipsoid zone are reportedly associated with poor visual acuity in chronic CSC [37, 38]. Further longitudinal studies are needed to investigate choroidal blood flow and these clinical findings.

The limitations of our study included the small sample size and short follow-up period. Moreover, we did not investigate the relationship between the choroidal blood flow measured by LSFG and increased permeability of the choroidal vessels obtained using ICGA. Hyperpermeability of choroidal blood vessels and the consequent increase in choroidal hydrostatic pressure are considered to be the key pathogenic factors for CSC. The association between choroidal blood flow and localised hyperpermeability of the choroid in the subretinal space may be an indicator of disease activity and requires further investigation.

## Data Availability

The datasets generated and/or analysed during the current study are not publicly available due to data privacy concerns but are available from the corresponding author on reasonable request.

## Abbreviations

CSC: Central serous chorioretinopathy
LSFG: Laser speckle flowgraphy
FA: Fluorescein angiography
ICGA: Indocyanine green angiography
1/2PDT: Half-dose photodynamic therapy
1/3PDT: One-third dose photodynamic therapy
SRD: Serous retinal detachment
SD-OCT: Spectral-domain optical coherence tomography
BCVA: Best-corrected visual acuity
CRT: Central retinal thickness
CCT: Central choroidal thickness
MBR: Mean blur rate
m-MBR: Macular MBR
ONH: Optic nerve head
IOP: Intraocular pressure
RPE: Retinal pigment epithelium
LogMAR: Logarithm of the minimum angle of resolution
CNV: Choroidal neovascularization
SRF: Subretinal fluid
OCT: Optical coherence tomography
EDI-OCT: Enhanced-depth imaging OCT
SBP: Systolic blood pressure
DBP: Diastolic blood pressure
MAP: Mean arterial pressure
OPP: Ocular perfusion pressure
